# Provision of dietary education in UK-based cardiac rehabilitation: a cross-sectional survey conducted in conjunction with the British Association for Cardiovascular Prevention and Rehabilitation

**DOI:** 10.1017/S0007114523002374

**Published:** 2024-03-14

**Authors:** Emily James, Tom Butler, Simon Nichols, Stuart Goodall, Alasdair F. O’Doherty

**Affiliations:** 1 Department of Sport, Exercise and Rehabilitation, Northumbria University, Newcastle-Upon-Tyne NE1 8ST, UK; 2 Diabetes Research Centre, University of Leicester, Leicester, UK; 3 National Institute for Health Research (NIHR) Leicester Biomedical Research Centre, University Hospitals of Leicester NHS Trust and the University of Leicester, Leicester, UK; 4 Faculty of Health, Social Care and Medicine, Edge Hill University, Ormskirk, UK; 5 Cardiorespiratory Research Centre, Edge Hill University, Ormskirk, UK; 6 School of Nursing, Midwifery and Paramedic Practice, Robert Gordon University, Aberdeen, UK; 7 Advanced Wellbeing Research Centre, Sheffield Hallam University, Sheffield, UK

**Keywords:** Cardiac rehabilitation, Dietetics, Education, Health service

## Abstract

Dietary education is a core component of cardiac rehabilitation (CR). It is unknown how or what dietary education is delivered across the UK. We aimed to characterise practitioners who deliver dietary education in UK CR and determine the format and content of the education sessions. A fifty-four-item survey was approved by the British Association for Cardiovascular Prevention and Rehabilitation (BACPR) committee and circulated between July and October 2021 via two emails to the BACPR mailing list and on social media. Practitioners providing dietary education within CR programmes were eligible to respond. Survey questions encompassed: practitioner job title and qualifications, resources, and the format, content and individual tailoring of diet education. Forty-nine different centres responded. Nurses (65·1 %) and dietitians (55·3 %) frequently provided dietary education. Practitioners had no nutrition-related qualifications in 46·9 % of services. Most services used credible resources to support their education, and 24·5 % used BACPR core competencies. CR programmes were mostly community based (40·8 %), lasting 8 weeks (range: 2–25) and included two (range: 1–7) diet sessions. Dietary history was assessed at the start (79·6 %) and followed up (83·7 %) by most centres; barriers to completing assessment were insufficient time, staffing or other priorities. Services mainly focused on the Mediterranean diet while topics such as malnutrition and protein intake were lower priority topics. Service improvement should focus on increasing qualifications of practitioners, standardisation of dietary assessment and improvement in protein and malnutrition screening and assessment.

A healthy diet is arguably the most controversial modifiable risk factor within cardiac rehabilitation (CR) for secondary prevention of CVD. Abundance of contradictory information on foods or specific nutrients, uncritical media coverage of some low-quality nutritional research studies and the limited quality of nutrition training of healthcare professionals, outside of dietetics, likely contribute to these controversies^(1–3)^.

Recent guidelines and recommendations from the American Heart Association^(4)^, British Association for Cardiovascular Prevention and Rehabilitation (BACPR)^(5)^ and European Society of Cardiology^(6)^ agree on the major components of a cardioprotective diet: plentiful and varied fruits and vegetables, wholegrains, lean sources of protein, limited processed food and alcohol and unsaturated fats as the predominant dietary fat source. These guidelines should help standardise dietary education in CR. However, in some cases, the evidence base is inconclusive or divisive.^(7–10)^ Furthermore, co-existing diabetes (20–50 % of people with CVD)^(11–14)^, obesity (40–70 % of people with CVD)^(15,16)^, chronic kidney disease (23–28 % of people with CVD)^(17,18)^, chronic obstructive pulmonary disease (11–35 % of people with CVD)^(19,20)^ and sarcopenia (18–35 % of people with CVD)^(21,22)^ add complexity to the nutritional needs of people living with CVD. Knowledge, understanding and guidelines for nutritional support in CR are continually developing.^(5)^ Therefore, skilled and knowledgeable practitioners are needed to provide dietary education. Globally, clinicians from multiple professions, including dietitians, nurses, doctors and nurse educators, deliver nutritional education or diet-related care as part of secondary prevention of CVD^(23)^, diabetes^(24,25)^ and non-alcoholic fatty liver disease^(26)^. However, which practitioners deliver dietary education in CR in the UK is currently unknown.

Evidence to support implementation of dietary guidelines in practice is often derived from randomised controlled trials, such as the Lyon Diet Heart Study^(27)^ or CORDIOPREV study^(28)^, and prospective cohort studies, including those previously reviewed^(29)^. It is important to understand how, and by whom, dietary education is provided in CR centres to ensure consistent, high-quality practice is being delivered across the UK. It is unknown whether guidelines are effectively implemented in clinical practice.

The aim of this cross-sectional survey was to: (1) characterise practitioners who provide dietary education in UK-based CR and (2) understand the format and content of dietary education in UK-based CR.

## Methods

### Study design

This voluntary, open, cross-sectional, electronic survey was uploaded to the Online Surveys platform (Jisc) and disseminated to UK-based CR providers in conjunction with the BACPR. The methods and results are reported in accordance with the Strengthening the Reporting of Observational Studies in Epidemiology – nutritional epidemiology (STROBE-nut) checklist^(30)^.

### Sample

Any practitioners responsible for providing dietary education at a UK-based CR programme, including early and long-term maintenance CR, were eligible to submit a survey response after providing informed consent. Practitioners working at CR centres outside of the UK, or without a dietary education component, were ineligible.

### Survey development

The survey content was developed by the research team, comprising academics and a dietitian, and approved by members of the BACPR elected council. The first page of the online survey outlined the purpose and demands of the study, inclusion and exclusion criteria, estimated time for completion and the researchers’ data management plan. Survey questions were designed to investigate: (1) the profession and qualifications of dietary education providers, (2) the resources that they use to inform the dietary advice they give to patients, (3) the content and delivery method of diet sessions and (4) and the extent to which educational content is individually tailored. The final version of the online survey can be found in online Supplementary Material 1.

Electronic information was stored on secure, password-protected Online Surveys and Northumbria University OneDrive accounts. The survey totalled seventeen pages, incorporating study information, screening and consent questions, and thirty-nine (thirty-seven mandatory) multiple choice and fifteen (nine mandatory) free-text response questions. For nineteen multiple choice questions, selection of ‘other’ as a response generated a mandatory free-text box. Non-response to mandatory questions prohibited progression to the next survey page. Survey progress was displayed throughout, and respondents could review and change their answers using the ‘Previous’ button on each page.

One response was permitted from each CR centre. To identify duplicate responses, participants confidentially provided the name and location of their CR centre. Duplicate responses were filtered using the following selection criteria: (1) dietitian responses were preferred, (2) the most complete response from each centre and (3) the earliest submission.

### Survey dissemination

Before dissemination, the functionality of the online survey was tested by the authors. The survey was open between 1st July 2021 and 31st October 2021 and distributed via email and social media platforms (Twitter) belonging to the authors and the BACPR. Recruitment emails (online Supplementary Material 2) were sent to 869 BACPR members via the BACPR mailing list on two occasions: 19th July 2021 and 1st October 2021. Social media posts included a brief description of the survey aim and target demographic, and a link to the survey site. No incentives were offered for participation.

### Data analysis

Responses collected via Online Survey were downloaded into commercial software (IMB SPSS Statistics, version 27.0). Most (85 %) survey questions were mandatory to avoid accumulation of partial datasets.

Categorical data are reported as frequency and percentage. Continuous data are reported as median with inter-quartile range (IQR), minimum and maximum values. Two non-mandatory tick-box questions asked practitioners to rank variables based on their inclusion in standard practice or identify them as ‘not applicable’. Where ranking questions were partially completed, the missing data were grouped with ‘not applicable’. Free-text responses submitted under the option ‘other’ for multiple choice questions were grouped with existing tick-box responses, where appropriate, or entered as their own category. Where free-text responses required a numerical value, and the respondent provided a range, the median value was taken. Uninterpretable or implausible responses were noted and removed. All other free-text responses were exported to NVivo V.12 Pro for enumerative content analysis.^(31)^ All responses were coded inductively^(32)^. Initial categories were visualised using the Nvivo Hierarchy Chart tool to identify emerging patterns. Overarching and sub-categories were then refined before re-coding the data into final categories^(32)^. The frequency and/or percentage that each category and sub-category features in the responses received are reported.

## Results

### Survey responses

**Fig. 1.**
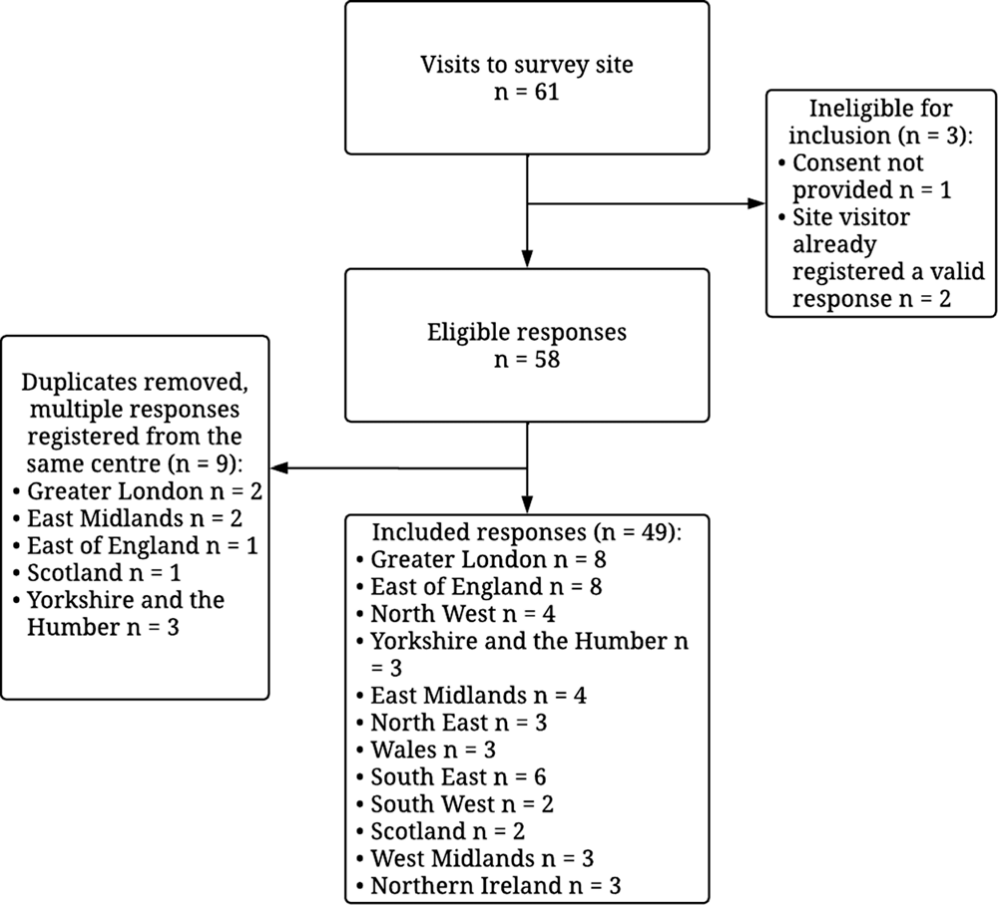
Schematic diagram of survey responses.

**Fig. 2.**
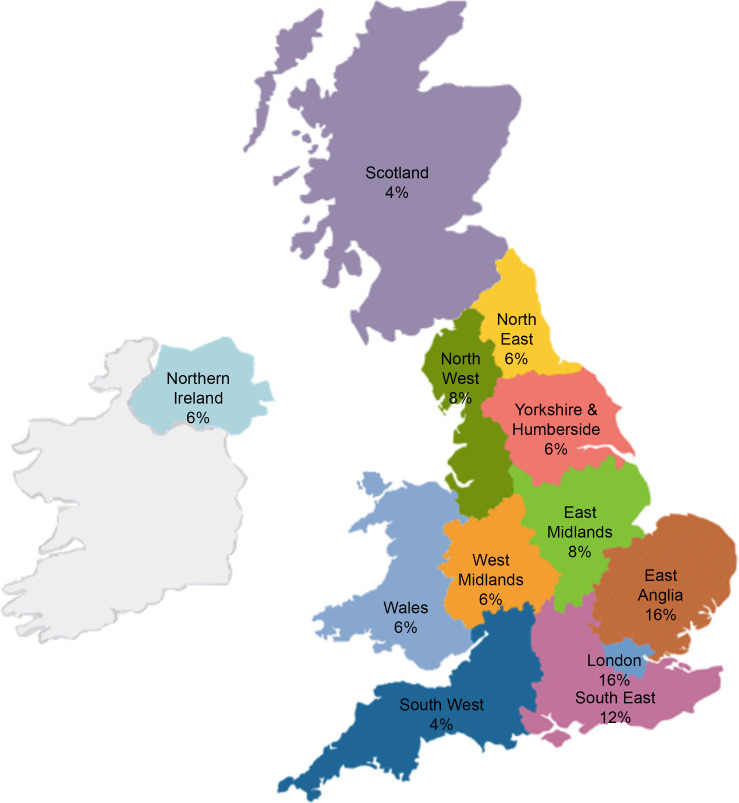
Distribution of CR centres across the UK, from which we received survey responses. CR, cardiac rehabilitation.

Sixty-one visits to the survey site were registered; 12 were ineligible/duplicate responses (Fig. 1). We analysed forty-nine survey responses. The included CR centres were located across all regions of the UK (Fig. 2) and primarily delivered early rehabilitation (*n* 38, 77·6 %), long-term maintenance rehabilitation (*n* 3, 6·1 %) or both equally (*n* 8, 16·3 %).

### Practitioner demographics

**Fig. 3.**
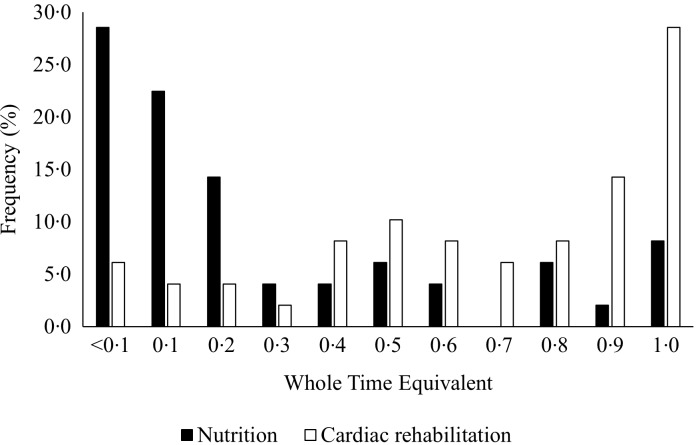
Distribution of working hours spent on nutrition (black bars) and cardiac rehabilitation (white bars) by practitioners in a typical working week using whole-time equivalents (WTE), where 0·1 equals half a day. Bars represent the frequency each WTE was selected as a response.

Survey responses were contributed by the following professionals who provided dietary education in UK CR: dietitians (*n* 11, 22·4 %), specialist nurses (*n* 23, 46·9 %), exercise professionals (*n* 8, 16·3 %), physiotherapists (*n* 5, 10·2 %), CR specialist (*n* 1, 2·0 %) and an assistant practitioner (*n* 1, 2·0 %). Most respondents were not solely responsible for providing dietary education at their CR centre (*n* 40, 81·6 %). According to those health professionals from which survey responses were taken on each centres behalf, dietary education was also being provided by a dietitian (*n* 19, 47·5 %), specialist nurse (*n* 14, 35·0 %), healthcare assistant (*n* 2, 5·0 %), physiotherapist (*n* 1, 2·5 %), CR practitioner (*n* 1, 2·5 %), paramedic (*n* 1, 2·5 %) and assistant practitioner (*n* 1, 2·5 %). Twenty-seven (55·1 %) CR centres employed at least one dietitian to deliver dietary education at their programme. Practitioners providing dietary education were employed under NHS pay-scale bands three (*n* 2, 4·1 %), four (*n* 4, 8·2 %), five (*n* 8, 16·3 %), six (*n* 31, 63·3 %), seven (*n* 31, 63·3 %) and eight (*n* 2, 4·1 %), where higher bands indicate seniority. One practitioner was self-employed (2·0 %) and two responded ‘not applicable’ (4·1 %). Dietitians were employed under NHS pay-scale bands five (*n* 5, 18·5 %), six (*n* 16, 59·3 %) and seven (*n* 6, 22·2 %). Three (6·1 %) respondents were unsure of their dietitian colleagues’ pay band. Considering their entire job scope, Fig. 3 shows the typical weekly distribution of time practitioners spent on CR and nutrition. 28·6 % of practitioners spent less than half a day per week (< 0·1 whole-time equivalent) on nutrition. 79·6 % of practitioners spent ≤ 0·5 whole-time equivalent on nutrition.

Around half of CR centres had practitioners with no formal nutrition-related qualifications providing dietary education (*n* 23, 46·9 %). Highest-level qualifications specifically related to nutrition were a BACPR 1-d course (*n* 13, 26·5 %), undergraduate (*n* 15, 30·6 %) or post-graduate (*n* 8, 16·3 %) degree in dietetics, undergraduate degree in nutrition (*n* 1, 2·0 %) or level three (*n* 5, 10·2 %), four (*n* 2, 4·1 %) or five (*n* 2, 4·1 %) nutrition-related courses. Ten (25·0 %) respondents were unsure of their colleagues’ highest level of nutrition-related qualification.

### Programme characteristics

CR programmes were hospital (*n* 5, 10·2 %), community (*n* 20, 40·8 %) or home based (*n* 3, 6·1 %). Others were a combination of hospital and community based (*n* 3, 6·1 %), community and home based (*n* 1, 2·0 %) or all three modes (*n* 17, 34·7 %). The median programme duration was 8 weeks (IQR 8, 10; range 2, 25; *n* 48). One response submitted 0 weeks, which was removed. Programmes involved one (*n* 26, 53·1 %), two (*n* 13, *n* 26·5 %), four (*n* 1, 2·0 %), six (*n* 5, 10·2 %) or seven (*n* 4, 8·2 %) sessions per week (median: 1, IQR 1, 2).

During the CR programme, dietary education was delivered to patients twice (median; *n* 45; IQR 1, 3, range 1, 12), including information delivered in person, and remote or manualised information. Four responses without a numerical value were removed. The median number of group-based dietary education sessions provided was one (IQR 0, 1). No group sessions were provided in thirteen CR programmes (26·5 %). Where group sessions were provided, the practitioner to patient ratio was 1:3 (*n* 1, 2·0 %), 1:4 (*n* 2, 4·1 %), 1:5 (*n* 5, 10·2 %), 1:6 (*n* 5, 10·2 %), 1:7 (*n* 1, 2·0 %), 1:8 (*n* 6, 12·2 %) or 1:10 (*n* 12, 26·5 %). There were six (12·3 %) invalid responses (online Supplementary Material 2). One-to-one sessions covering dietary education were provided in thirty-five (71·4 %) CR centres (median 1, IQR 0, 2; range 0, 12). Provision of one-to-one dietary education sessions was reported by a similar proportion of CR centres with (*n* 19. 70·4 %) and without (*n* 16, 72·7 %) a dietitian.

### Dietary assessment

General diet history was assessed at the start of CR at 39 centres (79·6 %), using general discussion with the patient about their dietary intake (*n* 13, 34·2 %), 24-h recall (*n* 12, 30·8 %), Mediterranean diet score or modified Mediterranean diet tool (*n* 10, 25·6 %), food diaries (*n* 7, 17·9 %), an undefined in-house assessment (non-specific responses submitted, such as ‘general assessment’, ‘diet assessment’ and ‘nurse assessment’; *n* 7, 17·9 %), 7-d recall (*n* 3, 7·7 %), FFQ (*n* 2, 5·1 %) or standardised questionnaires (*n* 2, 5·1 %). Presence or risk of malnutrition was not formally assessed in twenty-one programmes (49·2 %). Others identified malnutrition using the Malnutrition Universal Screening Tool (MUST; *n* 23, 46·9 %), changes to BMI or body mass (*n* 2, 4·1 %), in-house assessment by a dietitian (*n* 3, 6·1 %) and/or verbal discussion around eating patterns or appetite loss (*n* 2, 4·1 %).

Ten CR centres (20·4 %) did not assess diet history at the start of the CR programme. Barriers included lack of time (*n* 7, 70 %), insufficient staff (*n* 2, 20 %), prioritisation of other tasks (*n* 2, 20 %) and lack of practitioner training or knowledge in dietary assessment (*n* 3, 30 %). In four CR centres (40 %), dietitian assessment occurred later in the CR programme or patients were offered a referral to dietitian or other nutrition specialist.

### Dietary education content

**Fig. 4.**
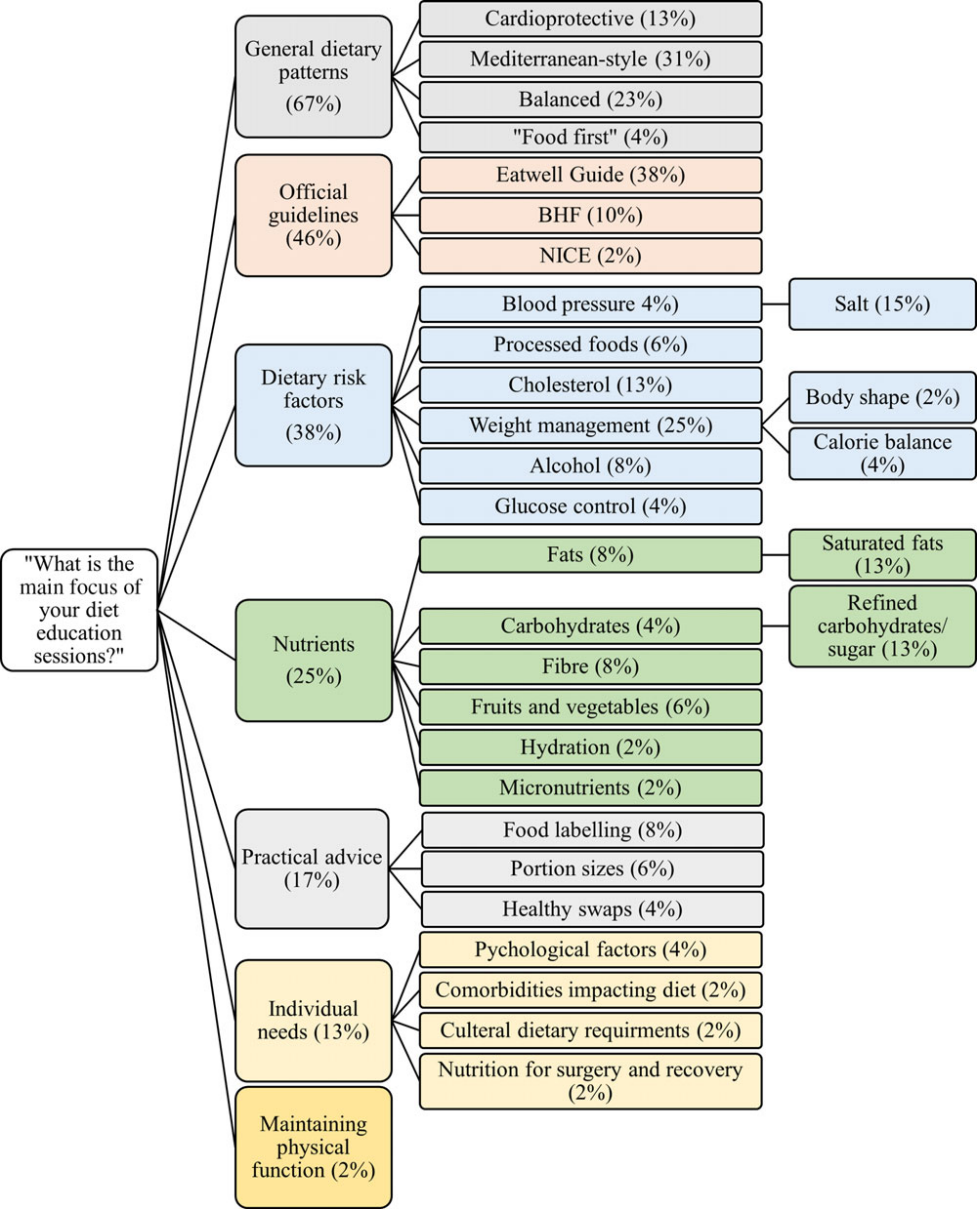
The key focus of dietary education in cardiac rehabilitation. Recommendations were categorised using enumerative content analysis of free-text responses. The frequency that each category features in the responses received are reported as percentage.

The delivery format and resources used in dietary education are presented in Table 1. All responses indicated that at least one resource was used during CR education sessions. Free-text responses (displayed as overarching and sub-categories) to the question ‘What is the main focus of your diet sessions?’ from forty-eight CR centres (98·0 %) are presented in Fig. 4.

**Table 1.** Format and delivery of, and resources used in, dietary education in CR (Numbers and percentages)

	*n*	%
Education delivery format		
Dedicated nutrition information	31	63·3
Nutrition information alongside other risk factor management strategies	18	36·7
Individualised or group-based information		
Individualised	14	28·6
Group based	7	14·3
Both	28	57·1
Resources used		
British Heart Foundation booklets	46	93·9
Practitioner-developed presentations or leaflets	36	73·5
The Eatwell Guide	34	69·4
National Health Service (NHS) website or weight management programmes	21	42·9
Visual aids	21	42·9
Quizzes or questionnaires	13	26·5
BACPR core competencies	12	24·5
Meal plans	11	22·4
Other resources	15	30·6
Free-text responses submitted with ‘Other’		
Recognised guidance, e.g., Diabetes UK, Flora, Heart UK, Change for Life and British Dietetic Association	7	14·3
Nutrition and diet resources	2	4·1
Cholesterol UK	1	2·0
Food diaries	1	2·0
Individualised plans	1	2·0
University graphics	1	2·0
Dean Ornish books	1	2·0
Public Health Collaborative	1	2·0
Diet doctor	1	2·0
How much dietary carbohydrate do you recommend to patients?		
Do not recommend a specific amount	37	75·5
∼40 % of total food intake (per the Eatwell Guide)	8	16·3
Other amount	4	8·2
Free-text responses submitted with ‘Other’		
Between five and eight portions daily	1	2·0
Between six and eight portions daily	1	2·0
Individualised based on HbA1c and fasting glucose levels	1	2·0
Individualised based on an unspecified variable	1	2·0
How much dietary fat do you recommend to patients?		
Do not recommend a specific amount	31	63·3
∼1 % of total food intake (per the Eatwell Guide)	6	12·2
Saturated fat < 10 % of energy intake (Joint British Societies guidance)	5	10·2
Total fat intake ≤ 30 % of total energy intake, and saturated fats ≤ 7 % of total energy intake (NICE primary care guidance)	5	10·2
Other amount	2	4·1
Free-text responses submitted with ‘Other’		
Low saturated fat (unspecified volume)	1	2·0
Recommendations based on BHF guidance	1	2·0
How much dietary protein do you recommend to patients?		
Do not recommend a specific amount	37	75·5
∼12 % of total food intake (per the Eatwell Guide)	6	12·2
Calculated relative to body mass (ranging between 0·8 and 2·0 g/kg/d (*n* 3); one response provided no numerical value)	4	8·2
Other amount	2	4·1
Free-text responses submitted with ‘Other’		
Calculated based on nitrogen content	1	2·0
Individualised based on an unspecified variable	1	2·0

### Recommending macronutrient intake

**Fig. 5.**
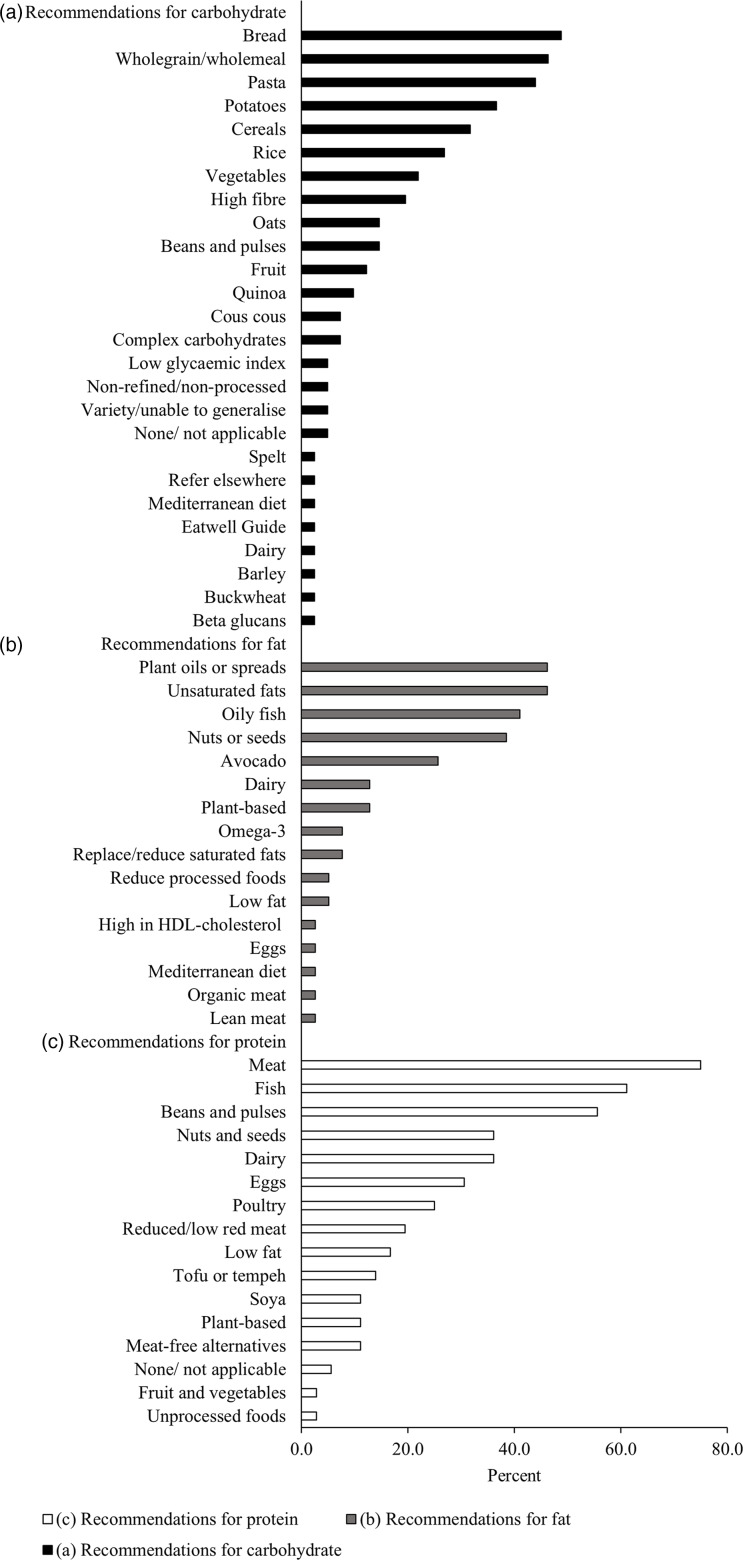
Sources of (a) carbohydrate (black bars), (b) fat (grey bars) and (c) protein (white bars) recommended to cardiac rehabilitation attendees by practitioners providing dietary advice. Recommendations were categorised using enumerative content analysis of free-text responses. The frequency that each category features in the responses received are reported as percentage.

Forty-one (83·7 %), thirty-nine (79·6 %) and thirty-seven (75·5 %) free-text responses were submitted describing the sources of carbohydrate, fat and protein most recommended to patients, respectively. After removal of one response to the protein sub-question, due to lack of clarity, responses were coded and quantified (Fig. 5).

Low carbohydrate diets were prescribed to patients at five CR centres (10·5 %) by modifying the diet to increase protein intake only (*n* 2, 40·0 %), increase fat and protein intake equally (*n* 1, 20·0 %) or by only focusing on decreasing carbohydrate intake (*n* 2, 40·0 %). Low-fat diets were prescribed to patients in eight CR centres (16·3 %) by modifying the diet to increase carbohydrate and protein content equally (*n* 2, 25·0 %) or by only focusing on decreasing fat intake (*n* 6, 75·0 %). No CR centres prescribe high-protein diets to their patients.

### Individualised dietary education

**Fig. 6.**
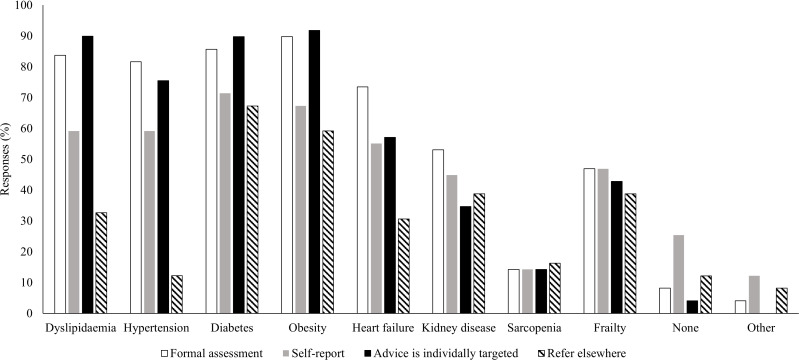
Practitioners providing dietary education as part of cardiac rehabilitation were asked to select all co-morbidities that apply to the following questions: (1) do you formally assess patients for any of the following co-morbidities that might affect the dietary advice you give them (white bars), (2) do you ask patients to self-report any of the following co-morbidities that might affect the dietary advice you give them (grey bars), (3) do you target individual dietary advice for patients based on any of the following co-morbidities (black bars) and (4) would you typically refer a patient to a specialist dietician for further input on individualised dietary considerations due to any of the following co-morbidities (striped bars). Free-text responses submitted under the option ‘other’ are detailed in online Supplementary Material 2.

Figure 6 shows whether co-morbidities are identified and/or targeted for individualised dietary advice. Tables 2 and 3 show motives for dietary modification discussed with, and dietary patterns offered to, patients as part of their standard CR programme, respectively. General health, maintaining a healthy weight and lowering cholesterol were all ranked highest, as reasons given for dietary modification (median rank 2, where 1 indicates key focus of dietary advice and 9 indicates least likely to be included in dietary advice). The Mediterranean-style diet was ranked as the most common dietary pattern offered to the typical patient (median rank 1).

**Table 2.** Practitioners indicated which motives for dietary modification are discussed with patients, reported as frequency (%)

			Rank																			
			1		2		3		4		5		6		7		8		9		N/A	
Reason for dietary modification	Frequency (%)		Frequency	%	Frequency	%	Frequency	%	Frequency	%	Frequency	%	Frequency	%	Frequency	%	Frequency	%	Frequency	%	Frequency	%
General health	46	93·9	23	46·9	4	8·2*	10	20·4	7	14·3	3	6·1	0		2	4·1	0		0		0	
Maintaining a healthy weight	47	95·9	22	44·9	7	14·3*	5	10·2	4	8·2	5	10·2	3	6·1	2	4·1	0		0		1	2·0
Lowering cholesterol	48	98·0	21	42·9	13	26·5*	5	10·2	2	4·1	4	8·2	0		2	4·1	2	4·1	0		0	
Lowering blood pressure	44	89·8	12	24·5	6	12·2	13	26·5*	6	12·2	4	8·2	2	4·1	2	4·1	0		0		4	8·1
Blood glucose control	46	93·9	12	24·5	7	14·3	9	18·4*	9	18·4	6	12·2	0		1	2·0	1	2·0	1	2·0	3	6·1
Maintaining physical function and independence	32	65·3	6	12·2	3	6·1	6	12·2	8	16·3*	2	4·1	3	6·1	6	12·2	3	6·1	3	6·1	9	18·3
Increasing life expectancy	25	51·0	7	14·3	2	4·1	3	6·1	4	12·2*	5	10·2	2	4·1	3	6·1	5	10·2	2	4·1	14	28·5
Maintaining bone health	25	51·0	7	14·3	1	2·0	3	6·1	2	4·1	7	14·3*	6	12·2	4	8·2	2	4·1	7	14·3	10	20·4
Maintaining muscle mass	27	55·1	5	10·2	2	4·1	6	12·2	4	8·2	5	10·2*	5	10·2	1	2·0	7	14·3	3	6·1	11	22·5
Other†	5	10·2																				

N/a, not applicable.

Taking a typical patient, practitioners ranked each motive for dietary modification in the order they feature in the advice given, where 1 = key focus and 9 = least likely to be included.

* Indicates median rank.

† Detailed in online Supplementary Material 2.

**Table 3.** Taking a typical patient, practitioners ranked common dietary pattern in the order they feature in the advice given, where 1 = most offered and 8 = least offered (Numbers and percentages)

	Rank																	
	1		2		3		4		5		6		7		8		N/A	
Dietary patterns	*n*	%	*n*	%	*n*	%	*n*	%	*n*	%	*n*	%	*n*	%	*n*	%	*n*	%
Mediterranean-style diet	36	73·5*	6	12·2	3	6·1	1	2·0	1	2·0	0		1	2·0	1	2·0	0	
Dietary approaches to Stop Hypertension (DASH)	4	8·2	11	22·4	8	16·3*	5	10·2	2	4·1	2	4·1	2	4·1	2	4·1	13	26·5
Low fat	7	14·3	8	16·3	11	22·4*	2	4·1	5	10·2	1	2·0	5	10·2	1	2·0	9	18·4
Low carbohydrate	1	2·0	5	10·2	11	22·4*	2	4·1	4	8·2	0		5	10·2	3	6·1	18	36·8
Plant based	0		6	12·2	5	10·2	4	8·2**	6	12·2	1	2·0	4	8·2	4	8·2	19	38·7
High protein	2	4·1	1	2·0	3	6·1	5	10·2	5	10·2*	2	4·1	2	4·1	4	8·2	25	51·0
Nordic diet	0		1	2·0	0		2	4·1	2	4·1	0		3	6·1*	5	10·2	36	73·4
Other†	2	4·1	0		0		0		0		0		1	2·0	1	2·0	45	91·8

N/a , not applicable.

* Indicates median rank.

** Median = 4·5.

† Detailed in online Supplementary Material 2.

**Fig. 7.**
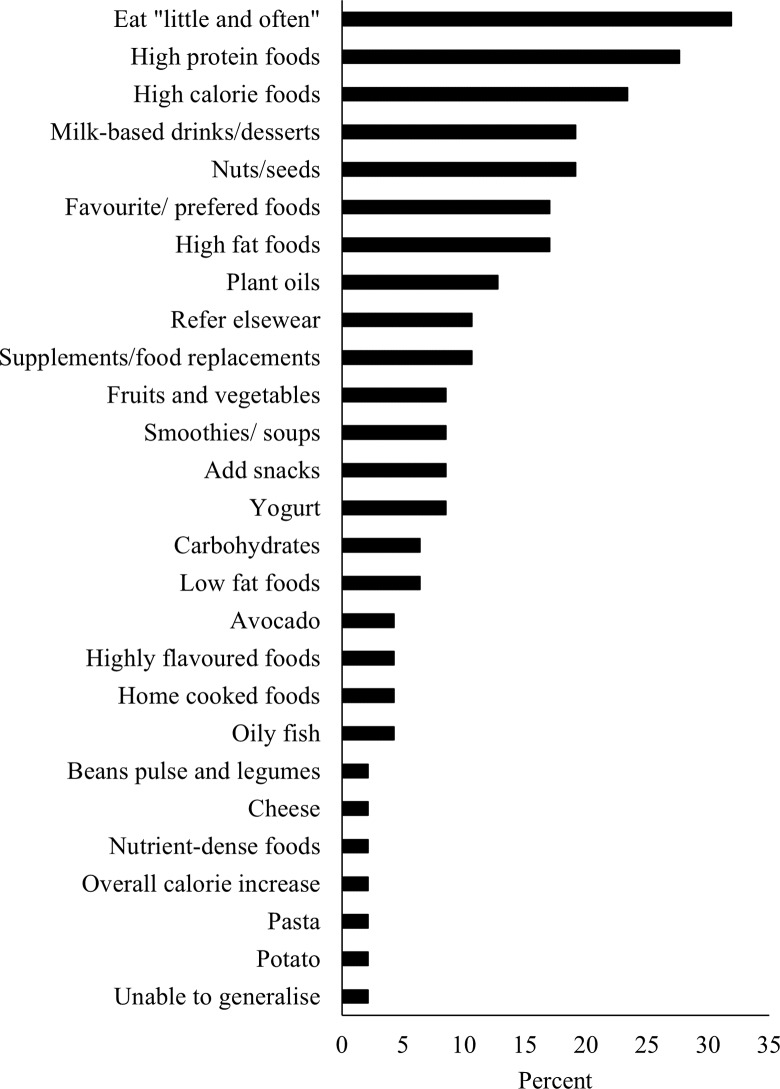
Dietary recommendations made to patients with poor appetite. Recommendations were categorised using enumerative content analysis of free-text responses. The frequency that each category features in the responses received are reported as percentage.

When promoting an energy deficit for weight loss, practitioners always (*n* 6, 12·2 %), sometimes (*n* 19, 38·8 %), rarely (*n* 7, 14·3 %) or never (*n* 17, 34·7 %) recommended maintaining high-protein intake. Dietary recommendations for patients with poor appetite are shown in Fig. 7, submitted as free-text responses by forty-seven (95·9 %) CR centres. Most commonly, practitioners recommend that patients eat ‘little and often’ (*n* 15, 31·9 %). Individualised recommendations for nutritional supplements were included as part of standard practice in twenty-two centres (44·9 %). Recommended supplements were oral nutritional support products (*n* 11, 50·0 %), vitamin D (*n* 3, 13·6 %), Ca (*n* 1, 4·5 %), Fe (*n* 1, 4·5 %), Mg (*n* 1, 4·5 %), antioxidants (*n* 1, 4·5 %), multivitamins (*n* 1, 4·5 %), protein (*n* 1, 4·5 %), unspecified type (*n* 3, 13·6 %) or referred elsewhere for advice (*n* 4, 18·2 %).

### Reviewing dietary changes

At forty-one (83·7 %) CR centres, compliance with dietary advice was followed up with patients. Forty (81·6 %) free-text responses specified that follow-ups were conducted via informal discussions (*n* 15, 37·5 %) and formally scheduled reviews (*n* 27, 67·5 %). Formal reviews occurred upon discharge of the patient (*n* 14, 35·0 %), at the programme midpoint (*n* 3, 7·5 %), upon progression to the next phase of CR (*n* 1, 2·5 %), one to two reviews over a 12-week CR programme (*n* 1, 2·5 %) and at week 6 out of 25 (*n* 1, 2·5 %). Some scheduled reviews were conducted by telephone (*n* 11, 27·5 %, including weekly or fortnightly (*n* 5, 12·5 %)), virtual clinic (*n* 1, 2·5 %) and email (*n* 1, 2·5 %). Additional methods of assessing dietary changes included reviewing achievement of previously set goals (*n* 5, 12·5 %), changes to blood lipids (*n* 3, 7·5 %), anthropometric measures (*n* 3, 7·5 %), the Mediterranean diet tool (*n* 1, 2·5 %) or a questionnaire 3 months after CR completion (*n* 1, 2·5 %). Five (12·5 %) responses stated that the occurrence, timing and method of follow-up appointments were individualised to each patient. In one (2·5 %) CR centre, patients were referred elsewhere for follow-up.

## Discussion

This cross-sectional survey aimed to understand provision of dietary education in UK-based CR programmes. The key findings were that (1) dietary education was provided by a dietitian (completely or partly) in 55 % of CR programmes but in nearly half of programmes at least one practitioner providing dietary education had no nutrition-related qualification, (2) dietary education predominantly focused around a Mediterranean-style diet, using information from the Eatwell Guide and the BHF, (3) dietary fat and carbohydrates were more commonly discussed with patients than protein and (4) dietary education was primarily aimed towards modifying weight, cholesterol levels and blood pressure but not muscle or bone health. These findings provide important insight into how UK-based CR centres deliver dietary education. This information should be used to inform future practice and potential development of new resources.

### Programme characteristics

According to the National Audit of Cardiac Rehabilitation (NACR), there were 231 CR providers across England, Northern Ireland and Wales in 2020^(33)^. Others reported a further sixty-nine CR centres in Scotland^(34)^. We received responses from forty-nine different CR centres. This is an estimated response rate of ∼16 % of eligible centres, although the total number of CR programmes in the UK is now likely lower after many programmes were suspended during the coronavirus disease 2019 pandemic^(35)^. Although our sample includes less than one-fifth of CR centres, we received responses from all regions across the UK (Fig. 2), representing a range of socio-economically diverse areas. Forty-one responses (84 %) were from England. This representation is consistent with data from the NACR^(33)^. The median 8-week duration of CR programmes in our sample was also consistent with NACR data^(33)^. Therefore, we are confident that key characteristics of the sample of CR programmes included in this survey are typical of CR centres across the UK.

### Dietary educators

Approximately half of the CR delivery teams in our sample contained dietary educators with no formal nutrition-related qualification. This is concerning and does not meet the BACPR minimum requirement for UK CR practitioners providing dietary education^(36)^. That is, practitioners should be appropriately qualified and skilled, with demonstrable knowledge of seven nutrition-specific competencies^(36)^. To our knowledge, this is the first study to report nutrition-specific qualifications of practitioners at UK-based CR programmes. Recently, European Society of Cardiology members recently cited lack of practitioner knowledge as detrimental to practise in secondary prevention of CVD^(37)^. Furthermore, 88 % of European Society of Cardiology members expected that greater patient compliance with lifestyle-related secondary prevention of CVD would be achieved with better education of the healthcare professionals advising them^(37)^.

A cardioprotective diet is a foundation for effective cardiovascular risk reduction^(38)^. Patients are more likely to achieve meaningful reductions in body mass and blood pressure if attending a CR programme with, compared to without, a nutrition component^(39)^. Importantly, dietary education delivered by a dietitian can significantly improve body composition and lower blood lipids and blood pressure, compared with education delivered by non-dietitian practitioners^(40,41)^. Despite this, around half of centres did not employ a dietitian and most practitioners delivering dietary education spend less than half a day per week focusing on nutrition (Fig. 3). Therefore, nutrition appears to be awarded low priority in UK CR programmes, despite it being a cornerstone of CVD prevention^(6,42)^.

The value to patients of access to appropriately qualified and skilled practitioners should not be understated. Our findings suggest that the nutritional component of CR should be urgently targeted for additional investment. Moreover, minimum qualification and/or competency requirements for the provision of dietary education could be more rigorously adhered to. The number of practitioners with nutrition-specific qualifications in UK CR should be increased.

### Dietary assessment

General dietary assessment was conducted with patients upon enrolment to CR in 80 % of programmes, adhering to the latest BACPR core standards^(43)^. However, dietary assessment methods varied. Practitioners who did not perform dietary assessments cited lack of time, training and knowledge in nutrition as barriers. According to current guidelines from the BACPR, dietary assessment should be undertaken with all patients^(43)^. Some dietary assessment tools in CVD have variable utility, due to low specificity to detect some nutrients^(44)^ or high time demand^(45)^. However, several validated rapid diet assessment tools exist^(46)^. Use of diet screening tools in practice can help standardise diet-related healthcare provision, identify patients in need of additional dietetic support and document changes to dietary habits following intervention^(46)^. In the CR setting, where dietary assessment is often performed by non-dietitians, validated scoring tools such as the Mediterranean Diet Score Tool^(47)^ can facilitate quick identification of patients in need of additional dietetic support. To improve the accuracy of reporting, pictorial guides to portion sizes can also be provided. If required, patients can then be referred to a registered dietitian for comprehensive assessment of their dietary history and individualised advice.

Malnutrition was assessed in around half of CR centres with the MUST tool^(48)^ being used most frequently (46·9 % of centres). Recently, one-third of patients undergoing percutaneous coronary intervention were at risk for malnutrition and lower nutritional risk score predicted in-hospital mortality^(49)^. Furthermore, poor nutrition status is a diagnostic domain in several definitions of frailty^(50)^, an age-related syndrome common in people with CVD^(49)^. Consensus and increased utilisation of malnutrition assessments are required, in addition to supported services once malnutrition is identified.

We found formally documented assessment of dietary intake of patients at the start and end of the programme was underutilised. A core component of UK CR is audit and evaluation^(51)^; however, this is precluded in centres who do not document dietary assessment. There is a need for standardising assessment and reassessment methods for dietary history, and appropriate training for performing these assessments, within UK CR.

### Dietary education

Dietary education was delivered twice (median) during CR programmes, although the frequency was highly varied across centres (between one and twelve sessions). Encouragingly, dietary education was most often in the form of dedicated nutrition content rather than in combination with other risk factor management strategies (Table 1), which could diminish the impact of the dietary education. Lara-Breitinger and colleagues^(52)^ suggest that ≥ 3 dietary education sessions are needed, based on a non-randomised controlled trial showing two education sessions were not better at modifying fat and carbohydrate intake compared with standard care after 6 weeks and 3 months.^(53)^ However, both intervention and standard care groups increased carbohydrate and reduced saturated fat intake, in line with recommended targets^(53)^. Therefore, dietary intake may be meaningfully altered with any dietary intervention. Where capacity allows, increasing the number of dietary education sessions in UK CR should be considered to optimise patient outcomes.

Figure 4 summarises the key focus of diet sessions delivered in CR. The Mediterranean-style diet was ranked as the pattern most frequently offered to patients (Table 2). Other whole-food approaches, including the DASH and Nordic diets, were offered. The basis of most healthy diet patterns, such as the DASH and Nordic diets and those advocated by the Eatwell Guide and BHF, shares key qualities with the Mediterranean diet^(54–57)^. Broadly, these recommendations are derived from research findings, which suggest a cardioprotective effect of eating plentiful fruits, vegetables^(58)^, legumes^(59)^, wholegrains^(60)^, olive oil and nuts^(61)^ and reducing or replacing saturated fats with unsaturated fats^(62)^. Although not widespread, there were non-evidence-based resources used in one CR programme, which is concerning. Reassuringly, dietary education appears largely evidence based. Centres should be signposted to evidence-based and alerted to non-evidence-based resources.

### Recommendations for macronutrient intake

Dietary education in UK CR appears to be focused on encouraging a general healthy dietary pattern rather than on individual nutrients. Reports from large cohort studies (*n* > 120 000) show that modulating macronutrient intake (either by altering food quantity or quality) is associated with changes to cardiovascular risk^(63)^. Substituting 5 % intake of carbohydrates from refined starches or added sugars, with PUFA or wholegrains, or substituting 5 % intake of SFA with PUFA reduced CVD risk in middle-aged and older adults^(63)^. Accordingly, in the current sample, wholegrains and unsaturated fats were favoured over non-wholegrain carbohydrate sources and saturated fats, respectively. However, 64 and 76 % of practitioners do not recommend a specific amount of dietary fat or carbohydrate to patients, respectively. This is consistent with guidelines advocating a ‘whole diet’ approach rather than focus on specific macronutrients^(5,42)^. In a UK Biobank study (*n* 210 106 adults aged 40–69 years), two-thirds exceeded recommended saturated fat intake and half did not meet the recommended carbohydrate intake^(64)^. Therefore, food-based recommendations should be tailored to encourage optimised fat and carbohydrate intake, without implementing specific nutrient targets.

Higher protein intake supports recovery without complications from illness or injury^(65)^ and is associated with higher muscle mass and strength in patients attending CR^(21)^. However, no responses referenced protein intake as a key focus of CR dietary education. When prompted, the most frequently cited examples of protein sources recommended to patients were meat products (81 %). The Eatwell Guide recommends non-animal sources of protein, in addition to animal sources, due to their greater environmental sustainability^(66)^. We found that beans, pulses and legumes were recommended by 61 % of practitioners, although soya products and meat-free alternatives such as mycoprotein (QuornTM) were less frequently mentioned. Additional focus on general protein intake and on non-animal sources of protein would be of benefit in CR dietary education.

### Tailoring dietary education for co-morbidities

Co-morbidities frequently addressed during CR included dyslipidaemia, hypertension, diabetes and obesity. These were addressed by formal assessment, individual targeting of dietary advice or referral to a specialised dietitian (Fig. 6). Practitioners more frequently referred patients to specialist dietitians for targeted dietary advice related to diabetes and obesity, over dyslipidaemia and hypertension. Access to tailored dietary information for a range of co-morbidities is important for people with CVD; among patients accessing CR programmes in the UK, 77·0 % presented with two or more co-morbidities^(33)^. It is promising that dietary education is tailored towards specific conditions in CR, either in-house or via referral to specialist dietitians. In a large, multi-country cohort study in people without history of CVD, presence of dyslipidaemia, hypertension, diabetes and abdominal obesity was associated with an increased risk of myocardial infarction over 10 years^(67)^. Modification of dietary intake to address these risk factors will likely improve long-term outcomes for patients following CR.

Sarcopenia and frailty were not prioritised by practitioners for assessment, targeting of individualised advice or referral to a specialised dietitian (Fig. 6). Furthermore, maintenance of muscle mass and bone health were low ranking reasons for dietary modification, as discussed with patients. This indicates that dietary modifications to support musculoskeletal health are low priority during current UK CR practice. This reflects our findings related to education on macronutrients, where protein intake was less commonly addressed than fat and carbohydrate intake. Importantly, the BACPR core standards highlight that dietary advice should support improvements in body composition, including gaining or maintaining muscle mass^(43)^. Sarcopenia and frailty should be considered within tailored nutrition prescriptions^(68)^.

### Implications for practice

Based on the findings of this survey, our recommendations for practice include

- The number of practitioners with nutrition-specific qualifications in UK CR should be increased, in accordance with the BACPR minimum competency requirements for the provision of dietary education.
- Standardised assessment and reassessment of dietary history should be implemented. Provision of appropriate training for practitioners to conduct these assessments is important.
- Consensus and increased utilisation of malnutrition assessments are required.
- Where capacity allows, increasing the number of dietary education sessions within core CR programmes should be considered.
- CR centres should be directed to evidence-based, over non-evidence-based, resources.
- Emphasis on overall protein intake, particularly from non-animal sources, would be of benefit in CR dietary education.
- Sarcopenia and frailty commonly coexist with CVD and should be considered within nutrition prescriptions, where indicated.

### Strengths and limitations

The study provides a novel cross-sectional overview of current dietary education in CR. We include responses representing all parts of the UK. Our findings set a platform from which dietary education within services can be targeted and improved.

The survey questions were designed by the research team, which we acknowledge as a potential source of bias. The geographical distribution and median duration of CR programmes from which we received responses were broadly aligned with national averages from the larger NACR sample. However, we acknowledge that self-selection bias will influence the findings of this voluntary survey and that a higher response rate would have improved the certainty of our findings. Survey recruitment methods predominantly targeted BACPR members, potentially limiting the representativeness of the sample. The survey was conducted during the coronavirus disease 2019 pandemic, which may have negatively affected the response rate and/or captured responses from CR programmes operating with an altered delivery method. Finally, responses were collated from one practitioner from each centre, who predominantly reported on their own experiences and practices. Clinicians who were not solely responsible for delivering dietary education at their CR centre might be unaware of the nature of dietary education being provided by their colleagues, as reported by others^(23,69)^.

### Conclusion

Dietary education content in UK CR overall reflects the current consensus of the cardioprotective diet. Encouragingly, known cardiovascular risk factors are routinely assessed and accounted for in tailored dietary advice. These findings are positive; however, standardisation of dietary assessment methods, education frequency, malnutrition screening and assessment and practitioner training would need prioritisation to ensure that consistent and comprehensive dietary education is being provided by appropriately skilled practitioners.

## Supporting information

James et al. supplementary material 1 James et al. supplementary material

James et al. supplementary material 2 James et al. supplementary material

## Supplementary material

For supplementary materials referred to in this article, please visit https://doi.org/10.1017/S0007114523002374

## References

[1] Freeman AM , Morris PB , Barnard N , et al. (2017) Trending cardiovascular nutrition controversies. J Am Coll Cardiol 69, 1172–1187. 28254181 10.1016/j.jacc.2016.10.086

[2] Freeman AM , Morris PB , Aspry K , et al. (2018) A clinician’s guide for trending cardiovascular nutrition controversies: part II. J Am Coll Cardiol 72, 553–568. 30049315 10.1016/j.jacc.2018.05.030

[3] Spector TD & Gardner CD (2020) Challenges and opportunities for better nutrition science—an essay by Tim Spector and Christopher Gardner. BMJ 369, m2470. 32591334 10.1136/bmj.m2470 PMC7318878

[4] Lichtenstein AH , Appel LJ , Vadiveloo M , et al. (2021) 2021 Dietary Guidance to Improve Cardiovascular Health: a scientific statement from the American Heart Association. Circulation 144, e472–e87. 34724806 10.1161/CIR.0000000000001031

[5] Butler T , Kerley CP , Altieri N , et al. (2020) Optimum nutritional strategies for cardiovascular disease prevention and rehabilitation (BACPR). Heart 106, 724–731. 32098809 10.1136/heartjnl-2019-315499 PMC7229899

[6] Visseren FLJ , Mach F , Smulders YM , et al. (2021) 2021 ESC Guidelines on cardiovascular disease prevention in clinical practice: developed by the Task Force for cardiovascular disease prevention in clinical practice with representatives of the European Society of Cardiology and 12 medical societies With the special contribution of the European Association of Preventive Cardiology (EAPC). Eur Heart J 42, 3227–3337. 36083202 10.1093/eurheartj/ehac458

[7] Schutz Y , Montani J-P & Dulloo AG (2021) Low-carbohydrate ketogenic diets in body weight control: a recurrent plaguing issue of fad diets? Obes Rev 22, e13195. 33471427 10.1111/obr.13195

[8] Wylie-Rosett J , Aebersold K , Conlon B , et al. (2013) Health effects of low-carbohydrate diets: where should new research go? Curr Diab Rep 13, 271–278. 23266565 10.1007/s11892-012-0357-5 PMC3595318

[9] Blesso CN & Fernandez ML (2018) Dietary cholesterol serum lipids, and heart disease: are eggs working for or against you? Nutrients 10, 426. 29596318 10.3390/nu10040426 PMC5946211

[10] Markey O , Vasilopoulou D , Givens D , et al. (2014) Dairy and cardiovascular health: friend or foe? Nutr Bull 39, 161–171. 25400508 10.1111/nbu.12086 PMC4207191

[11] Eser P , Marcin T , Prescott E , et al. (2020) Clinical outcomes after cardiac rehabilitation in elderly patients with and without diabetes mellitus: the EU-CaRE multicenter cohort study. Cardiovasc Diabetol 19, 37. 32192524 10.1186/s12933-020-01013-8 PMC7081600

[12] Soja AMB , Zwisler A-DO , Melchior T , et al. (2006) Prevalence and characteristics of impaired glucose metabolism in patients referred to comprehensive cardiac rehabilitation: the DANSUK study. Eur J Cardiovasc Prev Rehabil 13, 784–790. 17001219 10.1097/01.hjr.0000238391.12223.d0

[13] Lear SA , Ignaszewski A , Linden W , et al. (2002) A randomized controlled trial of an extensive lifestyle management intervention (ELMI) following cardiac rehabilitation: study design and baseline data. Curr Controlled Trials Cardiovasc Medicine 3, 9. 10.1186/1468-6708-3-9 PMC149404 12473163

[14] Lenzen M , Ryden L , Öhrvik J , et al. (2006) Diabetes known or newly detected, but not impaired glucose regulation, has a negative influence on 1-year outcome in patients with coronary artery disease: a report from the Euro Heart Survey on diabetes and the heart. Eur Heart J 27, 2969–2974. 17090612 10.1093/eurheartj/ehl363

[15] Kotseva K , Wood D , De Bacquer D , et al. (2016) EUROASPIRE IV: a European Society of Cardiology survey on the lifestyle, risk factor and therapeutic management of coronary patients from 24 European countries. Eur J Prev Cardiol 23, 636–648. 25687109 10.1177/2047487315569401

[16] Mehta RH , Bhatt DL , Steg PG , et al. (2008) Modifiable risk factors control and its relationship with 1 year outcomes after coronary artery bypass surgery: insights from the REACH registry. Eur Heart J 29, 3052–3060. 18996953 10.1093/eurheartj/ehn478 PMC2638656

[17] Cooper WA , O’Brien SM , Thourani VH , et al. (2006) Impact of renal dysfunction on outcomes of coronary artery bypass surgery. Circulation 113, 1063–1070. 16490821 10.1161/CIRCULATIONAHA.105.580084

[18] Reddan DN , Szczech LA , Tuttle RH , et al. (2003) Chronic kidney disease, mortality, and treatment strategies among patients with clinically significant coronary artery disease. J Am Soc Nephrol 14, 2373–2380. 12937316 10.1097/01.asn.0000083900.92829.f5

[19] Leavitt BJ , Ross CS , Spence B , et al. (2006) Long-term survival of patients with chronic obstructive pulmonary disease undergoing coronary artery bypass surgery. Circulation 114, I-430-I-4. 10.1161/CIRCULATIONAHA.105.000943 16820614

[20] Eriksson B , Lindberg A , Müllerova H , et al. (2013) Association of heart diseases with COPD and restrictive lung function – Results from a population survey. Respir Med 107, 98–106. 23127573 10.1016/j.rmed.2012.09.011

[21] Harada H , Kai H , Niiyama H , et al. (2017) Effectiveness of cardiac rehabilitation for prevention and treatment of sarcopenia in patients with cardiovascular disease - a retrospective cross-sectional analysis. J Nutr Health Aging 21, 449–456. 28346572 10.1007/s12603-016-0743-9 PMC12879826

[22] Kamiya K , Hamazaki N , Matsuzawa R , et al. (2017) Sarcopenia: prevalence and prognostic implications in elderly patients with cardiovascular disease. JCSM Clin Reports 2, 1–13.

[23] Mayr HL , Savill H , Law L , et al. (2023) ‘We work in silos’: exploring clinicians’ perspectives on the dietary management of coronary heart disease and type 2 diabetes in an Australian public hospital and community health service. Nutr Diet 80, 307–319. 36507592 10.1111/1747-0080.12789

[24] Gianfrancesco C & Johnson M (2020) Exploring the provision of diabetes nutrition education by practice nurses in primary care settings. J Hum Nutr Diet 33, 263–273. 31793070 10.1111/jhn.12720

[25] Parry Strong A , Lyon J , Stern K , et al. (2014) Five-year survey of Wellington practice nurses delivering dietary advice to people with type 2 diabetes. Nutr Diet 71, 22–27.

[26] Mayr HL , Kelly JT , Macdonald GA , et al. (2022) ‘Focus on diet quality’: a qualitative study of clinicians’ perspectives of use of the Mediterranean dietary pattern for non-alcoholic fatty liver disease. Br J Nutr 128, 1220–1230. 33766176 10.1017/S0007114521001100

[27] De Lorgeril M , Salen P , Martin J-L , et al. (1999) Mediterranean diet traditional risk factors, and the rate of cardiovascular complications after myocardial infarction. Circulation 99, 779–785. 9989963 10.1161/01.cir.99.6.779

[28] Delgado-Lista J , Alcala-Diaz JF , Torres-Peña JD , et al. (2022) Long-term secondary prevention of cardiovascular disease with a Mediterranean diet and a low-fat diet (CORDIOPREV): a randomised controlled trial. Lancet 399, 1876–1885. 35525255 10.1016/S0140-6736(22)00122-2

[29] Sofi F , Abbate R , Gensini GF , et al. (2010) Accruing evidence on benefits of adherence to the Mediterranean diet on health: an updated systematic review and meta-analysis. Am J Clin Nutr 92, 1189–1196. 20810976 10.3945/ajcn.2010.29673

[30] Lachat C , Hawwash D , Ocké MC , et al. (2016) Strengthening the Reporting of Observational Studies in Epidemiology–nutritional epidemiology (STROBE-nut): an extension of the STROBE statement. Nutr Bull 41, 240–251. 27587981 10.1111/nbu.12217 PMC4988500

[31] Grbich C (2012) Qualitative Data Analysis: An Introduction, 2nd ed. London, England: SAGE Publications.

[32] Braun V & Clarke V (2006) Using thematic analysis in psychology. Qual Res Psychol 3, 77–101.

[33] National Audit of Cardiac Rehabilitation (NACR) (2020) The National Audit of Cardiac Rehabilitation Quality and Outcomes Report 2020. British Heart Foundation. https://www.bhf.org.uk/-/media/images/information-support/publications/hcp/nacr_quality_and_outcomes_report_2020.pdf?rev=b2d2789a242a452ea43b128e8f02c11d (accessed March 2023).

[34] Turk-Adawi K , Supervia M , Lopez-Jimenez F , et al. (2019) Cardiac rehabilitation availability and density around the globe. EClinicalMedicine 13, 31–45. 31517261 10.1016/j.eclinm.2019.06.007 PMC6737209

[35] O’Doherty AF , Humphreys H , Dawkes S , et al. (2021) How has technology been used to deliver cardiac rehabilitation during the COVID-19 pandemic? An international cross-sectional survey of healthcare professionals conducted by the BACPR. BMJ Open 11, e046051. 10.1136/bmjopen-2020-046051 PMC8061561 33879492

[36] British Association for Cardiovascular Prevention and Rehabilitaton (BACPR) Diet Working Group (2019) Core Competences for the Diet Component: Healthy Eating and Body Composition For Cardiovascular Disease Prevention and Rehabilitation Services 2019. https://www.bacpr.org/__data/assets/pdf_file/0023/39434/633_BACPR_Dietetic_Competences_FINAL_2019.pdf (accessed Sept 2022).

[37] Fitzsimons D , Stępińska J , Kerins M , et al. (2020) Secondary prevention and cardiovascular care across Europe: a survey of European Society of Cardiology members’ views. Eur J Cardiovasc Nurs 19, 201–211. 31560214 10.1177/1474515119877999

[38] Piepoli MF , Hoes AW , Agewall S , et al. (2016) 2016 European guidelines on cardiovascular disease prevention in clinical practice. Eur Heart J, 37(29), 2315–2381. 27222591 10.1093/eurheartj/ehw106 PMC4986030

[39] Valentino G , Galgani J , Álamos M , et al. (2021) Anthropometric and blood pressure changes in patients with or without nutritional counselling during cardiac rehabilitation: a retrospective study. J Hum Nutr Diet 34, 402–412. 33098177 10.1111/jhn.12823

[40] Holmes AL , Sanderson B , Maisiak R , et al. (2005) Dietitian services are associated with improved patient outcomes and the MEDFICTS dietary assessment questionnaire is a suitable outcome measure in cardiac rehabilitation. J Am Diet Assoc 105, 1533–1540; quiz 49. 16183352 10.1016/j.jada.2005.08.001

[41] Riegel GR , Ribeiro PAB , Rodrigues MP , et al. (2018) Efficacy of nutritional recommendations given by registered dietitians compared to other healthcare providers in reducing arterial blood pressure: systematic review and meta-analysis. Clin Nutr 37, 522–531. 28065482 10.1016/j.clnu.2016.12.019

[42] National Institute for Health and Care Excellence (NICE) (2020) Recommendations | Acute Coronary Syndromes | Guidance: NICE. https://www.nice.org.uk/guidance/ng185/chapter/Recommendations#cardiac-rehabilitation-after-an-mi (accessed April 2023).

[43] British Association for Cardiovascular Prevention and Rehabilitaton (BACPR) (2023) The BACPR Standards and Core Components for Cardiovascular Disease Prevention and Rehabilitation 2023, 4th ed. London: BACPR.

[44] Neubeck L , Lowres N , Jackson A , et al. (2014) A simple screening tool for assessment of nutritional status in cardiac patients. Br J Cardiac Nurs 9, 508–512.

[45] Aberegg ES , Collins KK , Hinderliter JM , et al. (2020) Validity and reliability of a brief dietary assessment questionnaire in a cardiac rehabilitation program. J Cardiopulmonary Rehabil Prev 40, 280–283. 32604257 10.1097/HCR.0000000000000505

[46] Vadiveloo M , Lichtenstein AH , Anderson C , et al. (2020) Rapid diet assessment screening tools for cardiovascular disease risk reduction across healthcare settings: a scientific statement from the American Heart Association. Circ: Cardiovasc Qual Outcomes 13, e000094. 32762254 10.1161/HCQ.0000000000000094

[47] National Audit of Cardiac Rehabilitation (NACR) (2013) Mediterranean Diet Score Tool: NHS Digital. http://www.cardiacrehabilitation.org.uk/docs/Mediterranean-Diet-Score.pdf (accessed August 2023).

[48] Stratton RJ , Hackston A , Longmore D , et al. (2004) Malnutrition in hospital outpatients and inpatients: prevalence, concurrent validity and ease of use of the ‘malnutrition universal screening tool’ (‘MUST’) for adults. Br J Nutr 92, 799–808. 15533269 10.1079/bjn20041258

[49] Calvo E , Teruel L , Rosenfeld L , et al. (2019) Frailty in elderly patients undergoing primary percutaneous coronary intervention. Eur J Cardiovasc Nurs 18, 132–139. 30156426 10.1177/1474515118796836

[50] Giallauria F , Di Lorenzo A , Venturini E , et al. (2021) Frailty in acute and chronic coronary syndrome patients entering cardiac rehabilitation. J Clin Med 10, 1696. 33920796 10.3390/jcm10081696 PMC8071180

[51] British Association for Cardiovascular Prevention and Rehabilitation Exercise Professionals Group (BACPR-EPG) (2019) Essential Competencies and Minimum Qualifications Required to Lead the Supervised Exercise Component in (Early) Core Cardiac Rehabilitation. Position Statement 2019, version 3rd ed. London: The British Association for Cardiovascular Prevention and Rehabilitation.

[52] Lara-Breitinger K , Lynch M & Kopecky S (2021) Nutrition intervention in cardiac rehabilitation: a review of the literature and strategies for the future. J Cardiopulmonary Rehabil Prev 41, 383–388. 34727557 10.1097/HCR.0000000000000660

[53] Timin MT , Shores KV & Reicks M (2002) Behavior change outcomes in an outpatient cardiac rehabilitation program. J Am Dietetic Assoc 102, 664–671. 10.1016/s0002-8223(02)90152-5 12008991

[54] Sacks FM , Obarzanek E , Windhauser MM , et al. (1995) Rationale and design of the Dietary Approaches to Stop Hypertension trial (DASH): a multicenter controlled-feeding study of dietary patterns to lower blood pressure. Ann Epidemiol 5, 108–118. 7795829 10.1016/1047-2797(94)00055-x

[55] Adamsson V , Reumark A , Cederholm T , et al. (2012) What is a healthy Nordic diet? Foods and nutrients in the NORDIET study. Food Nutr Res 56, 18189. 10.3402/fnr.v56i0.18189 PMC3386552 22761599

[56] British Heart Foundation (BHF) (2019) Eat Better to Reduce Your Risk of Heart and Circulatory Diseases 2019. https://www.bhf.org.uk/informationsupport/publications/healthy-eating-and-drinking/eat-better (accessed March 2022).

[57] Scarborough P , Kaur A , Cobiac L , et al. (2016) Eatwell Guide: modelling the dietary and cost implications of incorporating new sugar and fibre guidelines. BMJ Open 6, e013182. 10.1136/bmjopen-2016-013182 PMC5223664 28003292

[58] Aune D , Giovannucci E , Boffetta P , et al. (2017) Fruit and vegetable intake and the risk of cardiovascular disease, total cancer and all-cause mortality—a systematic review and dose-response meta-analysis of prospective studies. Int J Epidemiol 46, 1029–1056. 28338764 10.1093/ije/dyw319 PMC5837313

[59] Ha V , Sievenpiper JL , de Souza RJ , et al. (2014) Effect of dietary pulse intake on established therapeutic lipid targets for cardiovascular risk reduction: a systematic review and meta-analysis of randomized controlled trials. Cmaj 186, E252–62. 24710915 10.1503/cmaj.131727 PMC4016088

[60] Aune D , Keum N , Giovannucci E , et al. (2016) Whole grain consumption and risk of cardiovascular disease, cancer, and all cause and cause specific mortality: systematic review and dose-response meta-analysis of prospective studies. BMJ 353, i2716. 27301975 10.1136/bmj.i2716 PMC4908315

[61] Estruch R , Ros E , Salas-Salvadó J , et al. (2018) Primary prevention of cardiovascular disease with a Mediterranean diet supplemented with extra-virgin olive oil or nuts. N Engl J Med 378, e34. 29897866 10.1056/NEJMoa1800389

[62] Jakobsen MU , O’Reilly EJ , Heitmann BL , et al. (2009) Major types of dietary fat and risk of coronary heart disease: a pooled analysis of 11 cohort studies. Am J Clin Nutr 89, 1425–1432. 19211817 10.3945/ajcn.2008.27124 PMC2676998

[63] Li Y , Hruby A , Bernstein AM , et al. (2015) Saturated fats compared with unsaturated fats and sources of carbohydrates in relation to risk of coronary heart disease: a prospective cohort study. J Am Coll Cardiol 66, 1538–1548. 26429077 10.1016/j.jacc.2015.07.055 PMC4593072

[64] Bennett E , Peters SAE & Woodward M (2018) Sex differences in macronutrient intake and adherence to dietary recommendations: findings from the UK Biobank. BMJ Open 8, e020017. 10.1136/bmjopen-2017-020017 PMC5922487 29691247

[65] Cawood AL , Elia M & Stratton RJ (2012) Systematic review and meta-analysis of the effects of high protein oral nutritional supplements. Ageing Res Rev 11, 278–296. 22212388 10.1016/j.arr.2011.12.008

[66] Buttriss JL (2016) The Eatwell Guide refreshed. Nutr Bull 41, 135–141.

[67] Yusuf S , Joseph P , Rangarajan S , et al. (2020) Modifiable risk factors, cardiovascular disease, and mortality in 155 722 individuals from 21 high-income, middle-income, and low-income countries (PURE): a prospective cohort study. Lancet 395, 795–808. 31492503 10.1016/S0140-6736(19)32008-2 PMC8006904

[68] Abreu A , Frederix I , Dendale P , et al. (2021) Standardization and quality improvement of secondary prevention through cardiovascular rehabilitation programmes in Europe: the avenue towards EAPC accreditation programme: a position statement of the Secondary Prevention and Rehabilitation Section of the European Association of Preventive Cardiology (EAPC). Eur J Prev Cardiol 28, 496–509. 33611459 10.1177/2047487320924912

[69] Meyer SB , Coveney J & Ward PR (2014) A qualitative study of CVD management and dietary changes: problems of ‘too much’ and ‘contradictory’ information. BMC Fam Pract 15, 25. 24495674 10.1186/1471-2296-15-25 PMC3916316

