# Supplementary material for: Provision of dietary education in UK-based cardiac rehabilitation: a cross-sectional survey conducted in conjunction with the British Association for Cardiovascular Prevention and Rehabilitation
Source: Br J Nutr. 2023 Oct 23;131(5):880–93. doi: 10.1017/S0007114523002374 (PMC10864998; doi:10.1017/S0007114523002374)
Supplement: James et al. supplementary material 1 — James et al. supplementary material [file S0007114523002374sup001.pdf]

Dietary Advice in UK-based CR

Page 1

Provision of Dietary Advice in UK-based Cardiac Rehabilitation: A Cross-Sectional Survey.

INVESTIGATORS: Emily James, Dr Simon Nichols, Dr Stuart Goodall, Dr Tom Butler, Dr Alasdair O'Doherty

The purpose of this information sheet is to provide you with sufficient information so that you can make an informed decision about whether to take part in our study and give your informed consent. Please read this document carefully and raise any issues that you do not understand with the investigator.

1.What is the purpose of the project?

Our knowledge, understanding and guidelines for nutritional support in cardiac rehabilitation is continually developing. It is good practice to capture the education provided by cardiac rehabilitation centres to highlight good practice and areas for improvement. This helps to ensure that consistent high-quality practice is being delivered across the UK.

We would like to understand the current provision of dietary advice in UK-based cardiac rehabilitation. This information might inform future standardised guidelines for dietary advice in cardiac rehabilitation.

2. Why have I been selected to take part?

You have been invited to take part as you are involved in the provision of dietary advice to cardiac patients at a UK-based cardiac rehabilitation centre (Phase 3 or 4). Ideally, we would like to receive one response per cardiac rehabilitation centre in the UK. This could be from a dietitian, specialist nurse, healthcare assistant, or any other healthcare professional delivering dietary advice to patients as part of their cardiac rehabilitation.

3. Do I have to take part?

No. Participation in this survey is voluntary, and you will not experience any penalty or loss of benefit if you do not take part. If you do decide to take part, you can end your involvement in the study at any time by exiting the webpage.

4. What will I have to do?

We would like you to complete a survey which will collect information about routine dietary advice given to cardiac patients within your cardiac rehabilitation service. The survey should take no more than 15 minutes.

5. What is the exclusion criteria (i.e. are there any reasons why I should not take part)?

You should not complete this survey if you do not currently provide dietary advice to cardiac patients as part of their standard cardiac

rehabilitation.

6. What are the possible benefits of taking part?

We anticipate that the information you provide in the survey could contribute to the development of new dietary advice standards and guidelines for cardiac rehabilitation programmes. This will be of benefit to practitioners and cardiac patients across the UK.

7. What are the possible risks / disadvantages of taking part?

Other than the small amount of time required to complete this survey, there are no foreseeable risks or disadvantages to taking part.

8. Will my participation involve any psychological discomfort or embarrassment?

No questions involving personal information are included in this questionnaire. Therefore, we do not expect there to be any psychological discomfort or embarrassment associated with taking part. You may leave any questions unanswered that you are not comfortable with and can end your involvement in the study at any time, without reason or prejudice, by exiting the webpage. If you have any concerns about this survey, please contact a researcher using the email addresses provided below.

9. Will be participation be kept confidential and anonymous?

Yes, survey responses will be anonymised. We will ask you to enter the name and location of your rehabilitation service at the start of the questionnaire, so that we do not accept multiple responses from the same cardiac rehabilitation centre. This information will only be available to the research team and will not be published with the findings. The results of the survey will be analysed by region, similar to the regions used in the National Audit of Cardiac Rehabilitation, and not by your specific centre.

Personal data will be treated in accordance with university guidelines and the Data Protection Act (2018).

10. Who will have access to the information that I provide?

Any information and data gathered during this research study will only be available to the research team. Should the research be presented or published in any form, then that information will be generalised (i.e., your personal information or data will not be identifiable).

11. How will my information be stored / used in the future?

Relevant conclusions formulated from the results will be used as part of a PhD thesis. The general findings might be reported in a scientific journal, or presented at a research conference, but they will always remain anonymous. All information and data gathered during this research will be stored in line with the Data Protection Act (2018). The data may be used by members of the research team only for purposes appropriate to the research question, but at no point will your personal information or data be revealed.

Survey responses will be collected through Online Survey, an online platform that meets Northumbria University's requirements for processing personal data. Electronic information will be stored on secure, password-protected Online Surveys and Northumbria University OneDrive accounts, meeting General Data Protection Regulation requirements.

12. Has this investigation received appropriate ethical clearance?

This study received full ethical approval from Northumbria University Health and Life Sciences Ethics Committee. If you require confirmation of this, please contact the chair of ethics using the details below:

Dr Claire Thornton

Department of Sport and Exercise Sciences, Northumbria University

Claire.thornton@northumbria.ac.uk

13. Will I receive any financial rewards for taking part?

You will not receive any financial rewards for taking part

14. How can I withdraw from the project?

Initially, please speak to a researcher if you have any concerns about this study. You can withdraw from this study at any point, without reason or prejudice, by exiting the webpage. Any responses you have given up to this point will be retained and used by the research team for its original purpose. If, for any reason, you wish to withdraw your data please contact the investigator within a month

of your participation. After this date, it might not be possible to withdraw your individual data as the results might already have been published. As all data are anonymous, your individual data will not be identifiable in any way.

15. If I require further information who should I contact and how?

Researcher: Emily.j.c.james@northumbria.ac.uk

Senior academic: alasdairodoherty@northumbria.ac.uk

I have read and understood the study information. I agree to take part in this study. ✱ *Required*

- Yes
- No

Name of your cardiac rehabilitation centre (this information will only be available to the research team to avoid accepting multiple responses from the same centre. Names will not be published with the findings). ✱ *Required*

Have you previously completed **this** questionnaire? ✱ *Required*

- Yes
- No

Thank you for your time. We do not believe we have your informed consent to take part in this study. If you selected No in error, you may return to the previous page and edit your answer.

Thank you for taking the time to complete this questionnaire previously. We only require one response per person. If you selected Yes in error, you may return to the previous page and edit your answer.

Please note - Questions in this survey relate to your standard cardiac rehabilitation provision. Please answer honestly. We are interested in current provision across the UK, rather than what you believe is the ‘correct’ or ‘best practice’ answer. We understand that not all questions will be relevant to all cardiac rehabilitation programmes. If you do not have an answer to some questions, we ask that you please state this or leave the box blank rather than exiting the survey, as this will still be useful information.

Where is your cardiac rehabilitation centre located?

Using whole-time equivalent (WTE), where 0.1 equals half a day, approximately how much of your job role is dedicated to:

Please don't select more than 1 answer(s) per row.

	<0.1	0.1	0.2	0.3	0.4	0.5	0.6	0.7	0.8	0.9	1.0
Nutrition	☐	☐	☐	☐	☐	☐	☐	☐	☐	☐	☐
Cardiac rehabilitation	☐	☐	☐	☐	☐	☐	☐	☐	☐	☐	☐

What is your job role / profession?

If you selected Other, please specify:

Which AfC band do you currently work under?

What is your highest-level qualification specifically **related to nutrition**?

If you selected Other, please specify:

Is dietary advice at your cardiac rehabilitation centre provided to cardiac patients by anyone other than yourself?

- ○ Yes
- ○ No

What is their role / profession?

If you selected Other, please specify:

Which AfC band do they currently work under?

What is their highest-level qualification specifically **related to nutrition**?

- Post-graduate (Master or PhD) in dietetics
- Undergraduate degree in dietetics
- Undergraduate degree in nutrition
- BACPR 1-day course
- Level 3 nutrition-related course
- Level 4 nutrition-related course
- Level 5 nutrition-related course
- No formal nutrition-related qualification
- Unsure
- Other

If you selected Other, please specify:

Does your programme primarily deliver:

- Early rehabilitation (previously Phase III)
- Long-term maintenance (previously Phase IV)
- Both equally

Is your cardiac rehabilitation programme:

- Home-based
- Community-based
- Hospital-based
- A mixture of hospital- and community-based
- A mixture of hospital-, community-, and home-based
- Other

If you selected Other, please specify:

How many weeks does your cardiac rehabilitation programme last?

Please enter a whole number (integer).

How many weekly sessions are included in your standard cardiac rehabilitation programme?

Do you take patients' diet history at the start of their cardiac rehabilitation programme?

- ○ Yes
- ○ No

How are patients diet history assessed? Select all that apply.

- ☐ 24-hour food recall
- ☐ Food frequency questionnaire (FFQ)
- ☐ Mediterranean diet score tool
- ☐ 3-day food diary
- ☐ 7-day food diary
- ☐ Other

If you selected Other, please specify:

Please explain the barriers to undertaking dietary assessment.

Your answer should be no more than 300 characters long.

How do you identify patients with, or at risk of, malnutrition?

If you selected Other, please specify:

Do you **formally assess** patients for any of the following comorbidities that might affect the dietary advice given to them? Select all that apply.

- ☐ Dyslipidaemia
- ☐ Hypertension
- ☐ Diabetes
- ☐ Obesity

- ☐ Heart failure
- ☐ Kidney disease
- ☐ Sarcopenia
- ☐ Frailty
- ☐ None
- ☐ Other

If you selected Other, please specify:

Do you ask patients to **self-report** any of the following comorbidities that might affect the dietary advice given to them? Select all that apply.

- ☐ Dyslipidaemia
- ☐ Hypertension
- ☐ Diabetes
- ☐ Obesity
- ☐ Heart failure
- ☐ Kidney disease
- ☐ Sarcopenia
- ☐ Frailty
- ☐ None
- ☐ Other

If you selected Other, please specify:

On how many occasions is dietary advice delivered to patients during standard cardiac rehabilitation, including sessions delivered by a healthcare professional, remote, or manualised information?

On occasions where dietary adivce is delivered to patients, is the information:

- Dedicated primarily to diet (for example a dedicated nutrition session led by a healthcare professional, or a booklet including only nutrition information)
- Presented alongside other risk factor management strategies

During standard duration cardiac rehabilitation, how many sessions delivered by a healthcare professional covering dietary advice are group sessions?

Please enter a whole number (integer).

What is the typical staff to patient ratio in your group diet sessions? (Staff member: patients)

If you selected Other, please specify:

During standard duration cardiac rehabilitation, how many sessions covering dietary advice are one-to-one sessions?

Please enter a whole number (integer).

Is the dietary information given to patients:

- Individualised
- Group-based
- Both

Which resources do you used to provide dietary advice to patients? Select all that apply.

- Eatwell Guide
- NHS website
- Presentations / leaflets made by staff members
- Visual aids
- Meal plans
- BACPR core competencies
- Quizzes / questionnaires
- BHF booklet
- No resources
- Other

If you selected Other, please specify:

What is the **main focus** of your diet sessions? For example, following the Eatwell Guide or other "balanced diet" guide, reducing saturated fat or calorie intake.

Your answer should be no more than 300 characters long.

Which reasons for dietary modification do you discuss with your patients? Select all that apply.

- General health
- Maintaining a healthy weight
- Lowering cholesterol
- Lowering blood pressure
- Maintaining muscle mass
- Maintaining physical function and / or independence
- Increasing life expectancy

- [ ] Maintaining bone health
- [ ] Blood glucose control
- [ ] Other

If you selected Other, please specify:

Taking a typical patient, please rank the following reasons for dietary modification in order of how much they feature in the advice you give, where 1 = key focus and 9 = least likely to be included. You do not need to select a rank for all answers if they are not applicable. For example, if you typically feature three of the following reasons for dietary modification, please rank them 1-3 and leave the rest blank.

Please don't select more than 1 answer(s) per row.

Please don't select more than 1 answer(s) in any single column.

	1	2	3	4	5	6	7	8	9
General health	☐	☐	☐	☐	☐	☐	☐	☐	☐
Maintaining a healthy weight	☐	☐	☐	☐	☐	☐	☐	☐	☐
Lowering cholesterol	☐	☐	☐	☐	☐	☐	☐	☐	☐
Lowering blood pressure	☐	☐	☐	☐	☐	☐	☐	☐	☐
Maintaining muscle mass	☐	☐	☐	☐	☐	☐	☐	☐	☐
Maintaining physical function and / or independence	☐	☐	☐	☐	☐	☐	☐	☐	☐
Increasing life expectancy	☐	☐	☐	☐	☐	☐	☐	☐	☐
Maintaining bone health	☐	☐	☐	☐	☐	☐	☐	☐	☐
Maintaining blood glucose	☐	☐	☐	☐	☐	☐	☐	☐	☐

Which dietary patterns are most commonly offered to patients? Please rank in order, where 1 = most common and 8 = least common. You do not need to select a rank for all answers if they are not applicable. For example, if you typically offer three of the following dietary patterns, please rank them 1-3 and leave the rest blank.

Please don't select more than 1 answer(s) per row.

Please don't select more than 1 answer(s) in any single column.

	1	2	3	4	5	6	7	8
Mediterranean style	☐	☐	☐	☐	☐	☐	☐	☐
Dietary Approaches to Stop Hypertension	☐	☐	☐	☐	☐	☐	☐	☐
Nordic	☐	☐	☐	☐	☐	☐	☐	☐
Plant-based	☐	☐	☐	☐	☐	☐	☐	☐
Low carbohydrate	☐	☐	☐	☐	☐	☐	☐	☐
Low fat	☐	☐	☐	☐	☐	☐	☐	☐
High protein	☐	☐	☐	☐	☐	☐	☐	☐

Other	☐	☐	☐	☐	☐	☐	☐	☐

If you selected Other, please specify:

When promoting weight loss, do you recommend that patients maintain high protein intake whilst in calorie deficit?

- Always
- Sometimes
- Rarely
- Never

Which foods do you recommend to patients with poor appetite?

Your answer should be no more than 300 characters long.

Do you recommend nutritional supplements to patients?

- Yes
- No

Which nutritional supplements do you most commonly recommend?

Your answer should be no more than 300 characters long.

Are nutritional supplement recommendations:

- Individualised
- Group-based
- Both

Do you target **individual** dietary advice for patients based on any of the following comorbidities? Select all that apply.

- [ ] Dyslipidaemia
- [ ] Hypertension
- [ ] Diabetes
- [ ] Obesity
- [ ] Heart failure
- [ ] Kidney disease
- [ ] Sarcopenia
- [ ] Frailty
- [ ] None
- [ ] Other

If you selected Other, please specify:

Would you typically refer a patient to a specialist dietician for further input on individualised dietary considerations due to any other following comorbidities? Select all that apply.

- [ ] Dyslipidaemia
- [ ] Hypertension
- [ ] Diabetes
- [ ] Obesity
- [ ] Heart failure
- [ ] Kidney disease
- [ ] Sarcopenia
- [ ] Frailty
- [ ] None
- [ ] Other

If you selected Other, please specify:

How much dietary carbohydrate do you recommend to patients?

- ○ Do not recommend a specific amount
- ○ _ grams per kg body mass per day
- ○ _ grams per day

- ~40% of total food intake (per the Eatwell Guide)
- Other

If you selected Other, please specify:

If you recommend dietary carbohydrate using **grams per kg body mass per day** or **grams per day**, please specify the amount here:

Can you briefly describe the sources of carbohydrate you most commonly recomment to patients?

Your answer should be no more than 300 characters long.

Do you prescribe low carbohydrate diets?

If yes, how?

How much dietary fat do you recommend to patients?

- Do not recommend a specific amount
- _ grams per kg body mass per day
- _ grams per day
- ~1% of total food intake (per the Eatwell Guide)
- JBS3 guidelines (saturated fat is <10% of total fat intake, replace saturated with polyunsaturated fat)
- NICE guidelines (total fat intake is ≤30% of total energy intake, and saturated fats are ≤7% of total energy intake)
- Other

If you selected Other, please specify:

If you recommend dietary fat using **grams per kg body mass per day** or **grams per day**, please specify the amount here:

Can you briefly describe the sources of fat you most commonly recommend to patients?

Your answer should be no more than 300 characters long.

Do you prescribe low fat diets?

If yes, how?

How much dietary protein do you recommend to patients?

- Do not recommend a specific amount
- _ grams per kg body mass per day
- _ grams per day
- ~12% of total food intake (per the Eatwell Guide)
- Other

If you selected Other, please specify:

If you recommend dietary protein using **grams per kg body mass per day** or **grams per day**, please specify the amount here:

Can you briefly describe the sources of protein you most commonly recommend to patients?

Your answer should be no more than 300 characters long.

Do you prescribe high protein diets?

If yes, how?

Do you follow-up whether dietary advice has been implemented by patients?

- ○ Yes
- ○ No

Can you briefly describe how and when you follow-up with patients whether dietary advice has been implemented?

Your answer should be no more than 300 characters long.

Final page

Thank you!

1. What was the purpose of the project?

The purpose of this study was to understand provision of dietary advice in cardiac rehabilitation. We want to understand whether dietary advice provision differs between geographical location, how it is affected by resource and staffing availability, and whether the dietary recommendations address comorbidities other than cardiovascular disease. This information might inform future dietary advice provision in UK-based cardiac rehabilitation.

2. How will I find out about the results?

A summary of the study findings will be sent to the BACPR for dissemination to rehabilitation services in their directory. You may also email Emily James for a copy of this summary (Emily.j.c.james@northumbria.ac.uk).

3. What will happen to the information I have provided / how will the results be disseminated?

Data will be stored securely and will remain confidential in accordance with the Data Protection Act (2018). Generalised data will form part of a PhD thesis. It may also be used for publication in a scientific journal or conference presentation in accordance with the purpose of the research, but in all cases confidentiality will be assured and personal data will not be identifiable.

4. Will I receive individual feedback?

Individual feedback is not normally provided.

5. Have I been deceived in any way during the project?

No

6. If I change my mind and wish to withdraw the information I have provided, how do I do this?

If, for any reason, you wish to withdraw your data please contact the investigator within a month of your participation stating your cardiac rehabilitation centre name and location. After this date, it might not be possible to withdraw your individual data as the results might already have been published. As all data are anonymous, your individual data will not be identifiable in any way.

If you have any concerns or worries concerning the way in which this research has been conducted please contact Dr Nick Neave (Faculty Director of Ethics and Chair of the Faculty Research Ethics Committee) via email at nick.neave@northumbria.ac.uk

Researcher contacts

Principle Investigator: Emily.j.c.james@northumbria.ac.uk

Senior Investigator: Alasdair.odoherty@northumbria.ac.uk

Key for selection options

4 - Where is your cardiac rehabilitation centre located?

- South East (England)
- Greater London
- North West (England)
- East of England
- West Midlands
- South West (England)
- Yorkshire and the Humber
- East Midlands

North East (England)
Northern Ireland
Scotland
Wales

6 - What is your job role / profession?

Dietitian
Specialist nurse
Healthcare assistant
Other

7 - Which AfC band do you currently work under?

Band 1
Band 2
Band 3
Band 4
Band 5
Band 6
Band 7
Band 8
Band 9

8 - What is your highest-level qualification specifically related to nutrition?

Post-graduate (Master or PhD) in dietetics
Undergraduate degree in dietetics
Undergraduate degree in nutrition
BACPR 1-day course
Level 3 nutrition-related course
Level 4 nutrition-related course
Level 5 nutrition-related course
No formal nutrition-related qualification
Other

9.a - What is their role / profession?

Dietitian
Specialist nurse
Healthcare assistant
Other

9.b - Which AfC band do they currently work under?

Band 1
Band 2
Band 3
Band 4
Band 5
Band 6
Band 7
Band 8
Band 9

13 - How many weekly sessions are included in your standard cardiac rehabilitation programme?

1
2
3
4

5
6
7

15 - How do you identify patients with, or at risk of, malnutrition?
Malnutrition Universal Screening Tool (MUST)
Mini Nutritional Assessment (MNA)
Mini Nutritional Assessment Short Form (MNA-SF)
Not usually formally assessed
Other

21 - What is the typical staff to patient ratio in your group diet sessions? (Staff member: patients)
1:2
1:3
1:4
1:5
1:6
1:7
1:8
1:9
1:10
Not applicable
Other

36 - Do you prescribe low carbohydrate diets?
Yes
No

39 - Do you prescribe low fat diets?
Yes
No

42 - Do you prescribe high protein diets?
Yes
No
