# Supplementary material for: Provision of dietary education in UK-based cardiac rehabilitation: a cross-sectional survey conducted in conjunction with the British Association for Cardiovascular Prevention and Rehabilitation
Source: Br J Nutr. 2023 Oct 23;131(5):880–93. doi: 10.1017/S0007114523002374 (PMC10864998; doi:10.1017/S0007114523002374)
Supplement: James et al. supplementary material 2 — James et al. supplementary material [file S0007114523002374sup002.docx]

Provision of dietary education in UK-based cardiac rehabilitation: A cross-sectional survey conducted in conjunction with the British Association for Cardiovascular Prevention and Rehabilitation

**Supplementary material 2**

Example study recruitment email sent to BACPR members.

Re: *Dietary advice in UK-based cardiac rehabilitation.*

Dear [name],

Our knowledge, understanding and guidelines for nutritional support in cardiac rehabilitation is continually developing. We would like to understand the current provision of dietary advice in UK-based cardiac rehabilitation. This information might inform future standardised guidelines for dietary advice in cardiac rehabilitation.

If you currently provide dietary advice to patients at a UK-based cardiac rehabilitation centre, we would be grateful if you could complete our survey using the link below. The survey will capture the content, delivery methods and resources used in your usual dietary advice provision as part of standard cardiac rehabilitation.

Full details of the survey are provided through the link below. It should take no longer than 15 minutes and can be completed on a desktop computer or a smart phone. The findings of the study will be disseminated through the BACPR, scientific publications and conferences, and will contribute to a PhD thesis at Northumbria University.

We really appreciate your time and hope that you can help support us in this study.

Please click here [insert hyperlink] to complete the survey.

Best wishes,

[name]

BACPR Scientific Officer

Example study recruitment Twitter post.

“Nutritional support is an important part of #CardicRehab. If you deliver dietary advice to patients in a UK-based cardiac rehab centre, please help us understand current provision by completing this short survey [insert hyperlink]. RTs appreciated”

Invalid survey responses

Where cardiac rehabilitation programmes included group-based dietary education sessions, we asked providers to select the typical practitioner to patient ratio. The following six responses were not considered valid: four responses (8.2%) indicated that ≥1 practitioner-led dietary education session was group-based but selected “not applicable” for the staff to patient ratio. Two responses (4.1%) indicated that no sessions were group-based but selected a staff to patient ratio of 1:4 or higher for diet sessions.

Free-text responses

**Figure 6** presents methods of identifying and addressing (through dietary support) common comorbidities among patients attending cardiac rehabilitation. Free-text responses submitted under the option “other” for formal assessment (blue bars; n = 2) were allergies and intolerances, cancer, thyroid disorders, obstructive sleep apnoea and inflammatory bowel disease. Free-text responses submitted under the option “other” for participant self-report (grey bars; n = 6) were allergies and intolerances, cancer, thyroid disorders, obstructive sleep apnoea and inflammatory bowel disease, mobility, functional capacity, osteoporosis, and psychosocial factors impacting food choices. Free-text responses submitted under the option “other” for referral to a specialist dietitian (yellow bars; n = 4) were inflammatory bowel disease, coeliac, nutritional deficiencies or any other conditions outside the practitioner’s usual practice.

**Table 2** Practitioners indicated which motives for dietary modification are discussed with patients, reported as frequency (%). Free-text responses submitted under “other reasons for dietary modification” included improving mental health, bowel function, sleep, energy, and post-surgery healing.

**Table 3** Taking a typical patient, practitioners ranked common dietary pattern in the order they feature in the advice given, where 1 = most offered and 8 = least offered. Free-text responses submitted under “other dietary patterns” included Keto, a diet based on whole natural food or alternatives guided by patients’ preference.
